# Hypoxic Pulmonary Vasoconstriction in Humans

**DOI:** 10.1155/2013/623684

**Published:** 2013-08-20

**Authors:** Priyadharshanan Ariyaratnam, Mahmoud Loubani, Alyn H. Morice

**Affiliations:** ^1^Department of Cardiothoracic Surgery, Castle Hill Hospital, Cottingham HU16 5JQ, UK; ^2^Department of Academic Medicine, Castle Hill Hospital, Cottingham HU16 5JQ, UK

## Abstract

Hypoxic pulmonary vasoconstriction is the elegant theory put forward more than six decades ago to explain regional variations in perfusion within the lung in certain animal species in response to localised restrictions in oxygenation. Although considerable progress has been made to describe the phenomenon at the macroscopic level and explain it at the microscopic level, we are far from a universal agreement about the process in humans. This review attempts to highlight some of the important evidence bases of hypoxic pulmonary vasoconstriction in humans and the significant gaps in our knowledge that would need bridging.

## 1. Introduction

Hypoxic Pulmonary Vasoconstriction (HPV), although regarded as a physiological process that preserves systemic oxygenation, is also regarded as a pathophysiological entity predisposing to heightened pulmonary artery tones and subsequent pulmonary hypertension [1].

Whilst the process is established in some species, it is far from conclusive in humans where the exact site and nature of the response to hypoxia are the subject of much controversy and debate.

Reflected in this, although thorough and extensive, is the domination of much of the published reviews concerning this phenomenon with animal models of the disease process with little in the way of human data [2].

In this review, we seek to concentrate on the existing evidence for HPV within humans looking at responses all the way from isolated human pulmonary cells to clinical studies in patients. 

## 2. Human Isolated Pulmonary Artery Smooth Muscle Cells

### 2.1. Acute Hypoxia

Pulmonary artery smooth muscle cells (PASMCs) are located across the full length of the pulmonary arterial tree from large pulmonary arteries to (albeit to a lesser degree) the smaller arterioles [3]. 

Isolated PASMC react to hypoxia without either the influence of the surrounding lung parenchyma or systemic transmitters. This is an important concept as it has led some researchers to postulate that the PASMC may be the oxygen sensor to varying oxygen tensions [4]. This concept, however, is fraught with problems not least because the pulmonary arteries, even the smaller precapillary arterioles, are distant from the alveolus where gas exchange occurs [5].

Despite this, the mechanisms underlying the hypoxic response in PASMCs continue to be intriguing.

Calcium plays an important role in this response. Intracellular calcium is increased in isolated human PASMCs subjected to acute hypoxia (<5 minutes).

For example, Tang et al., using a mixture of functional pharmacology and gene knockout techniques, have shown that this acute hypoxic-calcium increase in human PASMCs is dependent to a lesser degree on voltage-gated calcium channels (inhibition of which attenuated the hypoxic calcium-related increases by 30%) and to a greater degree on other transmembrane channels such as the transient receptor potential (TRP) channels (inhibition of which attenuated the response by 60%) [6]. Further to this, the subtype of TRP channel plays an important role. Inhibition of store-operated TRP channels such as TRPC1, which function dependently on depletion of intracellular calcium stores, attenuated the hypoxic-calcium response to lesser degree than TRPC6 channels which are ligand operated.

Meng et al. have shown that this hypoxic-calcium increase is inhibited by arachidonic acid (AA) which considerably attenuated the calcium increase [7]. Breakdown of that AA by Cyclo-oxygenase-2 (COX) enhanced the hypoxic-calcium response suggesting AA itself mediates the attenuation of the hypoxic-calcium response rather than its derivatives. Interestingly, one of the inhibitors of this AA mediated inhibition is diacylglycerol which coincidentally is a ligand of the TRPC6 channel possibly suggesting a link between AA and receptor-operated TRP channels in regulating the calcium response to hypoxia in human PASMCs.

Animal studies have confirmed the importance of TRP channels in both the acute and chronic hypoxia response regulation [8]. For example in chronic hypoxic exposure (3-4 weeks) models of pulmonary hypertension in mice, Xia et al. confirmed that in the presence of antagonists of the TRP channel TRPV4, there was a reduced vasoconstrictive response to certain stimulants such as serotonin [9]. The same group had previously found that TRPV4 in rat PA is the only channel upregulated by chronic hypoxia which was associated with enhanced TRPV4-dependent Ca^2+^ influx in PASMCs, and the appearance of an intravascular pressure-activated myogenic tone [10].

Other important messengers besides calcium are reactive oxygen species (ROS) developed in response to changes in oxygen tension. Mehta et al. have shown that under sustained hypoxic conditions (1–4 hours), ROS are decreased within human PASMCs [11]. They also noted that normoxic ROS synthesis is predominately mitochondrial in origin within these cells, suggesting that the mitochondria may play a central role in the regulation of the acute hypoxic response in these cells.

 Remarkably, they compared the response to human coronary artery smooth muscle cells which also showed a reduction in ROS, but, as we know, systemic arteries dilate to hypoxia in contrast to that suggested in their pulmonary cousins. Why this differential contractile response to a similar intracellular phenomenon exists within the same species is still a mystery. 

These human results contrast to some animal models of acute hypoxia that demonstrate an increase in, for example, superoxide release from Complex III of smooth muscle cells. These oxidant signals diffuse into the cytosol and trigger increases in intracellular calcium that cause acute hypoxic pulmonary vasoconstriction [12].

It must be noted, however, that the aforementioned experiments avoided testing PASMC contractions in isolation per se, seeking rather to establish the intracellular signalling responses. Hence one cannot confirm if the respective changes in messaging pathways manifested as a contraction or dilatation. This is important as many of the cultured cells have come from companies with little details in the manuscripts about the site of origin of these cells: be they from larger pulmonary conductance arteries or from smaller arterioles or a mixture. Larger conductance arteries, as we know from animal and human studies, respond differently to hypoxia compared to resistance arteries. Understandably, it is technically difficult to assess contractility in single cells, but there are methods that have been used in animal models of HPV including tension forces generated by cells grown on a flexible growth surface (polymerized polydimethyl siloxane) manifesting as wrinkles and distortions of the surface under the cells or from measurements of myosin light chain phosphorylation [13].

### 2.2. Chronic Hypoxia

The plot thickens when one moves away from acute hypoxia to more chronic hypoxic insults. Wu et al. have shown that although acute hypoxia (5–10 minutes) stimulates a reduction in ROS, chronic hypoxia (48 hours) actually increases ROS production in human PASMCs [14]. This points to a potential difference at the subcellular level response to acute and chronic hypoxia which may explain the phenotypical changes seen in acute hypoxia (vasoconstriction) and chronic hypoxia (vascular remodelling).

Key regulators of vascular remodelling in response to chronic hypoxia include the Rho GTPase family of proteins. They are involved in cell adhesion, migration, and proliferation. An aesthetic study by Wojciak-Stothard et al. demonstrated in human PASMCs that Rho B levels were significantly increased in acute hypoxia (30 minutes–4 hours) [15]. This increase coincided with an increase in cytoskeleton remodelling within human PASMCs (represented by an increase in stress fibre formation). The authors further showed that this increase could be mimicked in normoxic conditions by inducing overexpression of Rho B implying Rho B's significant role in this process.

 To cement the role played by the Rho GTPase family, Yu et al. have shown that Rho kinase A (ROCK) expression was increased in sustained periods of acute hypoxia (4–12 hours) in human PASMCs, implying hypoxia may affect the Rho A/ROCK pathway, implicit in smooth muscle proliferation which may account for the hypoxic pulmonary hypertension seen in chronic models secondary to remodelling [16].

It is worth noting that the degree of acute hypoxia (0% O_2_–5% O_2_) and definitions of acute hypoxia and chronic hypoxia are variable which may give rise to inconsistencies. Part of the problem for this is that what may be acute in some animals may be chronic in others, and this may account for the variability in human studies.

Hence future experiments may focus on establishing the actual differential contractile responses of isolated cells cultured from various sites of the pulmonary arterial tree with consistent degrees and durations of hypoxia. 

## 3. Human Isolated Pulmonary Artery Endothelial Cells

Isolated human pulmonary artery endothelial cells (PAECs) are a major source of Nitric Oxide (NO) production in the pulmonary circulation via endothelial Nitric Oxide Synthase (eNOS). Experiments looking at the effect of hypoxia on endothelial cells have predominately been concerning more prolonged hypoxic exposure (48 hours). Takemoto et al. have told a continuation of the Rho story by demonstrating this chronic hypoxia is associated with an increase in ROCK expression with a simultaneous *decrease* in eNOS mRNA and protein expression in human PAECs [17]. The fact that eNOS increased by blocking ROCK with its selective inhibitor, hydroxyfasudil, demonstrated that eNOS may be dependent on ROCK.

Ghrelin is known to have protective effects on endothelial cells which are notoriously fragile entities. Yang et al. have shown that hypoxia for 24 hours reduces human PAEC viability, and this is prevented by pretreatment with ghrelin [18]. Subanalysis revealed that ghrelin increased NO secretion and eNOS phosphorylation in hypoxic conditions.

As NO is a potent vasodilator, eNOS inhibition would logically suggest that the vasoconstrictive-vasodilation balance may be shifted in chronic hypoxia towards constriction by reducing NO production.

Despite this, Beleslin-Čokić et al. have shown that chronic hypoxia (48 hours) actually causes an *increase* in NO production within human PAECs [19]. However, consistent with the aforementioned data, they did show a decrease in eNOS. This increase in Nitric Oxide has been confirmed in another study by Krotova et al. which showed that hypoxia increased NO in human lung microvascular endothelial cells [20]. So where would this increase in NO come from? It appears that there is an increase in other NOS enzymes notably inducible NOS (iNOS). Thus, it appears that the body may try to compensate for the constrictive remodelling seen in PASMCs in response to chronic hypoxia by inducing the release of dilatory mediators such as NO from PAECs via iNOS.

With regard to proliferation of PAECs, in contrast to PASMCs which have been shown to proliferate under prolonged periods of hypoxia (2–7 days), Yu and Hales have shown that human PAECs do not proliferate under hypoxic conditions [21]. This implies that human PASMCs may thrive under hypoxic stimuli as opposed to human PAECs which succumb to their fragility under similar conditions.

Studies have also demonstrated that PAECs, under hypoxic conditions, increase their permeability [15]. Perivascular oedema may contribute to changes in lung vascular resistance, but this would be due to passive compression rather than active hypoxic vasoconstriction.

**Figure 1:**
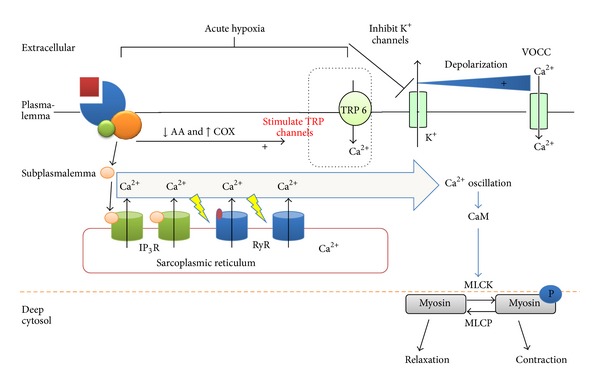
Mechanisms of acute hypoxic pulmonary vasoconstriction in human pulmonary artery smooth muscle cells.

**Figure 2:**
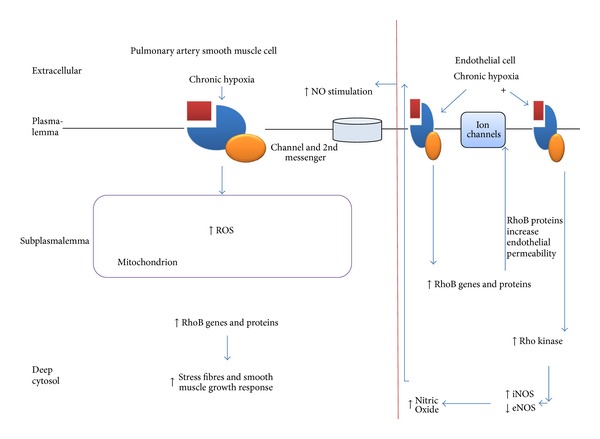
Vascular remodelling in response to chronic hypoxia in human pulmonary artery smooth cells and endothelial cells.

Figures 1 and 2 give an overview of the mechanisms by which acute and chronic hypoxia affect human pulmonary cells.

## 4. Human Pulmonary Artery Rings and Strips

Studies on human pulmonary artery strips (HPASs) and rings (HPARs) have been less consistent than studies on isolated cells. The majority of tissue was taken from healthy sections of lung from patients who underwent lobectomies for lung cancer. 

Hoshino et al. demonstrated that in HPASs, very little response occurred to acute hypoxic stimulation (<5 mins) when arteries (<5 mm diameter) were allowed to rest naturally with a tension of 2 g [22]. However, when arteries were prestimulated with histamine, they constricted to hypoxia. The response was significantly attenuated by compounds such as HA 1004 which is an inhibitor of cyclic nucleotide- and calmodulin-dependent protein kinases as well as attenuation by depletion of intracellular calcium. 

This necessity for prestimulation appears to be a common theme with HPARs as well. Demiryurek et al. have shown that precontracted rings constrict to acute hypoxia in a manner that is dependent on the presence of the endothelium as denuding the endothelium resulted in a markedly reduced hypoxic vasoconstrictive response [23].

However, Ohe et al. have shown that smaller HPARs (<2 mm) need not to have any degree of prestimulation and were able to constrict to hypoxia in their natural resting state in a calcium-dependent fashion [24].

We have shown that unstimulated larger HPAR (mean diameter 4 mm) dilate to hypoxia (0% O_2_) in a Nitric Oxide-independent manner and constrict to hyperoxia (95% O_2_) in a voltage-gated calcium-dependent fashion [25]. 

It is unclear why, therefore, a certain degree of prestimulation is warranted in some studies and not others particularly as we have just seen that human PASMCs have changes in intracellular calcium and other 2nd messenger components without recourse to prestimulation.

One reason for the variability of results could be because of the patients from whom the samples are taken from. These are lung cancer patients with varying degrees of other pulmonary and systemic disorders. Studies have shown there are considerable differences in the responsiveness of HPARs in patients with different pulmonary disorders. For example, Cases et al. showed that patients on bronchodilator therapies had greater contraction to noradrenaline and greater relaxation to acetylcholine compared to patients without bronchodilator requirements [26].

This appears to be supported by the fact that Pienado et al. have found that in patients with COPD (i.e. those on long-term bronchodilator therapy), there is an increase in the expression of certain potassium channels such as BK_Ca_ within HPARs which was positively correlated to a greater degree of constriction in response to hypoxia (again in the presence of preconstriction) [27].

It is apparent, therefore, that further work needs to be done on human pulmonary artery rings to elucidate if conditions other than prestimulation or preexisting lung disease (and associated pharmacological agents) play a role in varying the response to hypoxia.

## 5. Isolated Lung Models

One way in which the problems of site of HPV and isolated responses of the pulmonary circulation can be overcome would be with isolated perfused and ventilated human lung models. Although extensively researched in animals, isolated lung models have yet to be substantiated in humans. By ventilating the airways with varying concentrations of oxygen and monitoring airway and pulmonary artery pressures, one can investigate the overall pulmonary arterial response across the vascular tree without systemic cardiac output interference and the contribution of systemic hormonal effects.

A curious contribution to raised pulmonary arterial pressures may be the compression of the surrounding parenchymal tissue in response to hypoxia which has been demonstrated in animal and human studies [28]. By measuring changes in weight in the isolated lung and bronchial dilation, one could theoretically evaluate how much compressive effect oedema and bronchial pressures play, respectively, on the surrounding pulmonary vasculature.

An indirect evaluation of reaction to oxygen changes in the isolated lung has come as a byproduct of ex vivo lung perfusion (EVLP) strategies for donor lung optimisation prior to transplantation into patients. EVLP allows for improvement in lung physiology in lungs that would otherwise not be considered for transplantation in an age of limited donor supply. George et al. have shown that pulmonary artery pressures rise upon reperfusion of explanted lungs from patients in EVLP and that this rise is greatest when the initial period of ischaemia was greatest [29]. However, there is, as yet, little data on the effect of hypoxia reoxygenation via ventilating the explanted lung with varying degrees of oxygen, and this would be interesting to look at to evaluate the subsequent effect on pulmonary artery pressures.

## 6. Acute Hypoxic Challenges in Patients

Measurement of changes in pulmonary artery pressures in response to varying the inspired oxygen concentration (FIO_2_) in ventilated patients has yielded valuable results in the cumulative effect of hypoxia reoxygenation on both the pulmonary and systemic circulation.

Historically, in the 1950s and 1960s when a lot of the interest took off, there was actually a lot of conflicting evidence governing the effects of unilateral hypoxia (ventilating one lung with hypoxia and the other with normoxia or hyperoxia) on the pulmonary circulation. Fishman et al. from New York developed a method in 1955 combining bronchospirometry, with each lung breathing a specifically selected oxygen mixture, cardiac catheterization, and arterial cannulation to apply the Fick principle to measure blood flow within each lung as well as total blood flow in addition to pulmonary artery pressure in 6 anaesthetised male patients undergoing lung resection [30]. They found that by controlling one lung with a hyperoxic FIO_2_ (25–33%) and subjecting another to normoxia followed by hypoxia (10–12%) for 25 minutes, there was *no* alteration in blood flow to either lung or any changes in pulmonary vascular pressures.

 This is in contrast to Defares et al. from Sweden, who in 1958 utilised a similar technique but this time in 12 normal subjects, and they found that the blood flow to the hypoxic lung fell from 55% to 33% during a similar period and that concentration of hypoxia from Fishman's study as well [31]. They reasoned that this discrepancy between results could be attributed to the fact that Fishman used patients diagnosed with tuberculosis or suspected bronchogenic carcinomas whilst Defares' patients were healthy volunteers.

Defares' group later repeated the experiment in the lateral decubitus position as opposed to the subject in the supine position (as in the case of the previous experiments). They showed that this hypoxic redistribution of blood flow is not powerful to overcome the gravitational effects of blood diversion from the upper lung to the lower lung in the lateral thoracotomy position [32].

One of the leaders in this experimental field, Hedenstierna, compared flows in the hypoxic (FIO_2_ = 8%–12%) lung to the contralateral hyperoxic (FIO_2_ = 100%) lung in patients and found that although there was a significant reduction in the relative blood flow to the hypoxic lung (without a change in total cardiac output), there was no change in the pulmonary artery pressure [33]. Interestingly, they found that giving one lung hyperoxia and another normoxia made no difference to relative lung flows and pulmonary artery pressures which contradicts some animal models demonstrating hyperoxic vasodilation and others implying the oxygen-free radical release from hyperoxia may stimulate vasoconstriction [34, 35].

The results of regional blood flow redistribution have been repeated by Morrell et al. without cardiac output studies and without recourse to general anaesthesia but using radio-labelled isotopes and scintigraphic lung imaging under local anaesthetic conditions by utilising bronchoscopic techniques of brief periods of selective lobar occlusion [36]. The results have been similar to general anaesthetic studies. However, a potential confounding factor is that the partial pressure of carbon dioxide increased in the occluded lobe/segment, and this may contribute to a vasoconstrictive response in addition to the regional hypoxia.

From the aforementioned experiments, there is an obvious implication that reduced regional perfusion in hypoxia equates to a hypoxic vasoconstriction although this has not been demonstrated directly in these studies.

With regard to modulators of this hypoxic response, as the majority of these studies have been done by anaesthetists, they had a preconditioned disposition to investigate the effect of anaesthetic reagents on this redistribution effect. Hedenstierna's group had measured regional pulmonary blood flow in response to unilateral hypoxia in the presence of *clinical* doses of the maintenance anaesthetic agent isofluorane (1% and 1.5%) and found it had no effect on the hypoxic redistribution of blood flow [37].

One cannot help but be sceptical from the previous data with regard to a local modulation of HPV. If blood flow is indeed redistributed in response to hypoxia with hypoxic segments and normoxic/relatively hyperoxic segments dilating, then there must be some degree of central control either within the pulmonary circulation or within the body at large. However, contrary to this theory of a compensatory vasodilation response in the ventilated/hyperoxic lung would be the notion that the normal healthy human lung has a negligible resting tone and hence would not be able to dilate further as evident by the lack of vasodilatory response to inhaled Nitric Oxide in those subjects breathing air [38].

With regard to global hypoxia, a study by Talbot et al. showed that if patients received global hypoxia for 4 hours via a hyperbaric chamber without anaesthesia, there was an increase in the tricuspid pressure gradient as measured by echocardiography [39]. Tricuspid pressure gradient is a validated measure of pulmonary vascular tone although it is dependent on numerous factors not least the requirement for a certain degree of tricuspid regurgitation. Nevertheless, this small study consisting of 9 patients seems to demonstrate that global hypoxia, as opposed to regional hypoxia, would cause a net increase in pulmonary vascular tone. 

Cargill and Lipworth, using a similar method of measuring changes in pulmonary vascular tone, found that rendering healthy volunteers globally hypoxic for *brief periods* (30 minutes) by inhaling hypoxic gas mixtures, the tricuspid pressure gradient increased [40]. This increase was significantly attenuated by infusing patients with brain natriuretic peptide but not atrial natriuretic peptide prior to the hypoxic challenge.

This rise in PVR in response to global hypoxia appears to be supported by a similar study by Dorrington et al. in which 6 healthy volunteers received a more prolonged period of global hypoxia 5–8 hours in a hyperbaric chamber, measuring pulmonary vascular resistance (PVR) in a more invasive fashion utilising a pulmonary artery catheter [41]. They found that PVR increased more than twofold within a couple of hours of hypoxic exposure, and this was reversed upon normoxia.

Frostell et al., in awake healthy subjects, demonstrated that global inhalation of a hypoxic gas mixture for only 6 minutes resulted in an increase in the mean pulmonary artery pressure [38]. Importantly, however, this was accompanied by a significant increase in cardiac output, implying that HPV may not be the only response to hypoxia but there is a systemic cardiac response which also contributes to the raised pulmonary artery pressures (PAPs) as a consequence of hypoxia. Frostell also found that this rise in PAPs was attenuated by Nitric Oxide although whether this is antagonising HPV or merely vasodilating independently remains to be elucidated.

Global hypoxia appears therefore to impact on pulmonary artery pressures to a greater degree than regional hypoxia and oxygenated districts of the lung can compensate for suspected HPV as well whilst global hypoxia, it would seem, by a mixture of increased cardiac output (systemic control), and raised pulmonary tone (pulmonary control) shifts the balance towards reversible pulmonary hypertension. 

## 7. Studies on Chronic Airway Disease Patients

It is widely believed that the pulmonary hypertension associated with chronic hypoxia is due more to vascular remodelling, hypervolaemia, polycythaemia, and increased blood viscosity rather than HPV per se. The initial famous observations documented by Penaloza and Arias-Stella demonstrated that although Peruvians in general are born with right ventricular hypertrophy and elevated resting pulmonary, artery pressures, those who remain at sea level demonstrate a rapid reversal of these phenomenon whilst those remaining at high altitudes show little regression of these characteristics [42, 43]. Autopsy of these individuals revealed that this PAH was likely due to the thickening of the muscular layers of the pulmonary arterial tree. They measured partial pressures and saturations in these inhabitants and concluded there was a direct causal relationship between hypoxia and PAH.

Although this is an important finding, it may be an associative phenomenon rather than a causal one. For example, it is not as straightforward as this as oxygen concentration is not the only change upon moving to higher altitudes, there are changes in other atmospheric and ecological factors. Further to this, other humans who live in high altitudes such as Tibetans demonstrate neither raised pulmonary artery pressures nor any structural abnormalities of the pulmonary arterial tree [44]. This difference may be due to evolutionary factors as Tibetans have populated the high altitudes for a much longer time, and are therefore much better adapted to the resultant hypoxic conditions, as compared to Peruvians. However, this explanation still remains hypothetical.

Studies in patients with chronic lung disease have demonstrated possible existence of HPV controlling regional lung blood flow. For example, Santos et al. have shown that in patients with chronic obstructive pulmonary disorder (COPD) the dispersion of blood flow improved dramatically upon administration of 100% oxygen, the authors thereby stipulating that the HPV preexisting in these patients was alleviated [45]. Although this is a derivative finding that HPV exists in these patients, it is interesting to note that even in the chronic stages of hypoxia, HPV appears to be at least partially reversible.

Another interesting group of patients are those with obstructive sleep apnoea (OSA). Boyson et al. demonstrated that patients who have episodes of apnoea during the night have associated rises in pulmonary artery pressures, and this was in company of small fluctuations in oxygen saturations [46]. However, OSA patients have pulmonary hypertension during the daytime as well when they are not apnoeic [47], suggesting hypoxia associated with periods of apnoea is not a simple answer to the rises in PAP but other complex physiological and structural factors may be involved.

## 8. Conclusions and Future Directions

Hypoxic Pulmonary Vasoconstriction is a peculiar phenomenon where rather than the standard negative feedback mechanisms in place in the systemic circulation to improve oxygen delivery in times of scarcity, the lung seeks rather to shut things down completely. Animal studies have provided the basis to investigate basic and complex pathways that may explain this entity.

However, there are emerging inconsistencies. For example, there has been a preoccupation with the oxygen sensing mechanism residing in the normally hypoxic pulmonary artery when it is actually miles away (relatively) from the alveolus where gas exchange occurs. Recent animal studies have provided insights into the sensory apparatus living in the capillary-alveolar network which would make more logical sense, and this should stimulate human research into this area [5].

In addition, as there are significant interspecies differences in the responses of the pulmonary arterial tree to hypoxia and some species contradict HPV completely [48], there needs to be a greater drive to build upon the existing valuable human data to pinpoint the exact response of human pulmonary arteries to hypoxia and under what conditions.

 The two main problems with this are firstly there is a scarcity of human tissue and the centres that can obtain tissue from surgery are not necessarily the centres with the cutting-edge technology to investigate the samples. Secondly, we have seen that the variation in the response to oxygen even within humans may be due to the differences between “healthy” patients and those with significant pulmonary disease; invasive investigations of healthy subjects would present many ethical and logistical considerations [49]. Hence, those exemplary researchers with valuable expertise in animal models of HPV and technological methods must be allowed to liaise with clinicians who have access to the primary patient samples. 

In conclusion, despite the advancements made in discerning HPV in nature, the mechanisms behind the cellular, tissue, organ, and whole body response to hypoxia in humans remain in its infancy. 

## References

[1] Preston IR (2007). Clinical perspective of hypoxia-mediated pulmonary hypertension. *Antioxidants and Redox Signaling*.

[2] Sylvester JT, Shimoda LA, Aaronson PI, Ward JPT (2012). Hypoxic pulmonary vasoconstriction. *Physiological Reviews*.

[3] Kay JM (1983). Comparative morphologic features of the pulmonary vasculature in mammals. *American Review of Respiratory Disease*.

[4] Michelakis ED, Archer SL, Weir EK (1995). Acute hypoxic pulmonary vasoconstriction: a model of oxygen sensing. *Physiological Research*.

[5] Wang L, Yin J, Nickles HT (2012). Hypoxic pulmonary vasoconstriction requires connexin 40-mediated endothelial signal conduction. *The Journal of Clinical Investigation*.

[6] Tang C, To WK, Meng F, Wang Y, Gu Y (2010). A role for receptor-operated Ca^2+^ entry in human pulmonary artery smooth muscle cells in response to Hypoxia. *Physiological Research*.

[7] Meng F, To WKL, Gu Y (2008). Inhibition effect of arachidonic acid on hypoxia-induced [Ca^2+^]_i_ elevation in PC12 cells and human pulmonary artery smooth muscle cells. *Respiratory Physiology and Neurobiology*.

[8] Yang X-R, Lin M-J, Sham JSK (2010). Physiological functions of transient receptor potential channels in pulmonary arterial smooth muscle cells. *Advances in Experimental Medicine and Biology*.

[9] Xia Y, Fu Z, Hu J (2013). TRPV4 channel contributes to serotonin-induced pulmonary vasoconstriction and the enhanced vascular reactivity in chronic hypoxic pulmonary hypertension. *American Journal of Physiology. Cell Physiology*.

[10] Yang X-R, Lin AHY, Hughes JM (2012). Upregulation of osmo-mechanosensitive TRPV4 channel facilitates chronic hypoxia-induced myogenic tone and pulmonary hypertension. *American Journal of Physiology. Lung Cellular and Molecular Physiology*.

[11] Mehta JP, Campian JL, Guardiola J, Cabrera JA, Weir EK, Eaton JW (2008). Generation of oxidants by hypoxic human pulmonary and coronary smooth-muscle cells. *Chest*.

[12] Waypa GB, Marks JD, Guzy RD (2013). Superoxide generated at mitochondrial complex III triggers acute responses to hypoxia in the pulmonary circulation. *American Journal of Respiratory and Critical Care Medicine*.

[13] Murray TR, Chen L, Marshall BE, Macarak EJ (1990). Hypoxic contraction of cultured pulmonary vascular smooth muscle cells. *American Journal of Respiratory Cell and Molecular Biology*.

[14] Wu W, Platoshyn O, Firth AL, Yuan JX-J (2007). Hypoxia divergently regulates production of reactive oxygen species in human pulmonary and coronary artery smooth muscle cells. *American Journal of Physiology. Lung Cellular and Molecular Physiology*.

[15] Wojciak-Stothard B, Zhao L, Oliver E (2012). Role of RhoB in the regulation of pulmonary endothelial and smooth muscle cell responses to hypoxia. *Circulation Research*.

[16] Yu L, Quinn DA, Garg HG, Hales CA (2011). Heparin inhibits pulmonary artery smooth muscle cell proliferation through guanine nucleotide exchange factor-H1/RhoA/Rho kinase/p27. *American Journal of Respiratory Cell and Molecular Biology*.

[17] Takemoto M, Sun J, Hiroki J, Shimokawa H, Liao JK (2002). Rho-kinase mediates hypoxia-induced downregulation of endothelial nitric oxide synthase. *Circulation*.

[18] Yang D, Liu Z, Zhang H, Luo Q (2013). Ghrelin protects human pulmonary artery endothelial cells against hypoxia-induced injury via PI3-kinase/Akt. *Peptides*.

[19] Beleslin-Čokić BB, Čokić VP, Wang L (2011). Erythropoietin and hypoxia increase erythropoietin receptor and nitric oxide levels in lung microvascular endothelial cells. *Cytokine*.

[20] Krotova K, Patel JM, Block ER, Zharikov S (2010). Hypoxic upregulation of arginase II in human lung endothelial cells. *American Journal of Physiology. Cell Physiology*.

[21] Yu L, Hales CA (2011). Hypoxia does neither stimulate pulmonary artery endothelial cell proliferation in mice and rats with pulmonary hypertension and vascular remodeling nor in human pulmonary artery endothelial cells. *Journal of Vascular Research*.

[22] Hoshino Y, Obara H, Kusunoki M, Fujii Y, Iwai S (1988). Hypoxic contractile response in isolated human pulmonary artery: role of calcium ion. *Journal of Applied Physiology*.

[23] Demiryurek AT, Wadsworth RM, Kane KA, Peacock AJ (1993). The role of endothelium in hypoxic constriction of human pulmonary artery rings. *American Review of Respiratory Disease*.

[24] Ohe M, Ogata M, Katayose D, Takishima T (1992). Hypoxic contraction of pre-stretched human pulmonary artery. *Respiration Physiology*.

[25] Ariyaratnam P, Loubani M, Bennett RT (2013). hyperoxic vasoconstriction of human pulmonary arteries: a novel insight into acute ventricular septal defects. *ISRN Cardiology*.

[26] Cases E, Vila JM, Medina P, Aldasoro M, Segarra G, Lluch S (1996). Increased responsiveness of human pulmonary arteries in patients with positive bronchodilator response. *British Journal of Pharmacology*.

[27] Peinado VI, París R, Ramírez J, Roca J, Rodriguez-Roisin R, Barberà JA (2008). Expression of BKCa channels in human pulmonary arteries: relationship with remodeling and hypoxic pulmonary vasoconstriction. *Vascular Pharmacology*.

[28] Kapanci Y, Assimacopoulos A, Irle C (1974). ’Contractile interstitial cells’ in pulmonary alveolar septa: a possible regulator of ventilation/perfusion ratio? Ultrastructural, immunofluorescence, and in vitro studies. *Journal of Cell Biology*.

[29] George TJ, Arnaoutakis GJ, Beaty CA (2012). A physiologic and biochemical profile of clinically rejected lungs on a normothermic *ex vivo* lung perfusion platform. *Journal of Surgical Research*.

[30] Fishman AP, Himmelstein A, Fritts Jr HW, Cournand A (1955). Blood flow through each lung in man during unilateral hypoxia. *The Journal of Clinical Investigation*.

[31] Defares JG, Lundin G, Arborelius M, Stromblad R (1960). Effect of %unilateral hypoxia’ on pulmonary blood flow distribution in normal subjects. *Journal of Applied Physiology*.

[32] Arborelius M, Lundin G, Svanberg L, Defares JG (1960). Influence of unilateral hypoxia on blood flow through the lungs in man in lateral position. *Journal of Applied Physiology*.

[33] Hambraeus-Jonzon K, Bindslev L, Mellgård ÅJ, Hedenstierna G (1997). Hypoxic pulmonary vasoconstriction in human lungs: a stimulus-response study. *Anesthesiology*.

[34] Freeman BA, Topolosky MK, Crapo JD (1982). Hyperoxia increases oxygen radical production in rat lung homogenates. *Archives of Biochemistry and Biophysics*.

[35] Tate RM, Morris HG, Schroeder WR, Repine JE (1984). Oxygen metabolites stimulate thromboxane production and vasoconstriction in isolated saline-perfused rabbit lungs. *Journal of Clinical Investigation*.

[36] Morrell NW, Nijran KS, Biggs T, Seed WA (1995). Magnitude and time course of acute hypoxic pulmonary vasoconstriction in man. *Respiration Physiology*.

[37] Carlsson AJ, Bindslev L, Hedensierna G (1987). Hypoxia-induced pulmonary vasoconstriction in the human lung. The effect of isoflurane anesthesia. *Anesthesiology*.

[38] Frostell CG, Blomqvist H, Hedenstierna G, Lundberg J, Zapol WM (1993). Inhaled nitric oxide selectively reverses human hypoxic pulmonary vasoconstriction without causing systemic vasodilation. *Anesthesiology*.

[39] Talbot NP, Balanos GM, Dorrington KL, Robbins PA (2005). Two temporal components within the human pulmonary vascular response to *∼*2 h of isocapnic hypoxia. *Journal of Applied Physiology*.

[40] Cargill RI, Lipworth BJ (1995). Acute effects of ANP and BNP on hypoxic pulmonary vasoconstriction in humans. *British Journal of Clinical Pharmacology*.

[41] Dorrington KL, Clar C, Young JD, Jonas M, Tansley JG, Robbins PA (1997). Time course of the human pulmonary vascular response to 8 hours of isocapnic hypoxia. *American Journal of Physiology. Heart and Circulatory Physiology*.

[42] Penaloza D, Sime F, Banchero N, Gamboa R (1962). Pulmonary hypertension in healthy man born and living at high altitude: fifth Aspen Lung Conference: normal and abnormal pulmonary circulation. *Medicine Thoracic*.

[43] Arias-Stella J, Recavarren S (1962). Right ventricular hypertrophy in native children living at high altitude. *The American Journal of Pathology*.

[44] Groves BM, Droma T, Sutton JR (1993). Minimal hypoxic pulmonary hypertension in normal Tibetans at 3,658 m. *Journal of Applied Physiology*.

[45] Santos C, Ferrer M, Roca J, Torres A, Hernández C, Rodriguez-Roisin R (2000). Pulmonary gas exchange response to oxygen breathing in acute lung injury. *American Journal of Respiratory and Critical Care Medicine*.

[46] Boysen PG, Block AJ, Wynne JW (1979). Nocturnal pulmonary hypertension in patients with chronic obstructive pulmonary disease. *Chest*.

[47] Chaouat A, Weitzenblum E, Krieger J, Oswald M, Kessler R (1996). Pulmonary hemodynamics in the obstructive sleep apnea syndrome. Results in 220 consecutive patients. *Chest*.

[48] Wiener CM, Banta MR, Dowless MS, Flavahan NA, Sylvester JT (1995). Mechanisms of hypoxic vasodilation in ferret pulmonary arteries. *American Journal of Physiology. Lung Cellular and Molecular Physiology*.

[49] Miller FG (2003). Clinical research with healthy volunteers: an ethical framework. *Journal of Investigative Medicine*.

